# Antiproliferative and Antioxidative Bioactive Compounds in Extracts of Marine-Derived Endophytic Fungus *Talaromyces purpureogenus*

**DOI:** 10.3389/fmicb.2018.01777

**Published:** 2018-08-03

**Authors:** Madhuree Kumari, Sidhartha Taritla, Ankur Sharma, C. Jayabaskaran

**Affiliations:** Department of Biochemistry, Indian Institute of Science, Bangalore, India

**Keywords:** endophytes, *Talaromyces*, anticancer, antioxidative, parameter optimization, apoptosis

## Abstract

Endophytic fungi are now recognized as sources of pharmacologically beneficial, novel bioactive compounds. This study was carried out to evaluate antiproliferative and antioxidative potential of a seaweed endophytic fungus *Talaromyces purpureogenus*. Extracts with different solvents of the fungus grown on different liquid media were assayed for the antiproliferative and antioxidative activities. Tested 6 cancer cell lines, the highest antiproliferative activity was observed in ethyl acetate extract of total culture grown in Potato Dextrose Broth for 28 days in a dose-dependent manner. The highest antioxidative activity was observed in hexane extract of fungal culture grown in Malt Extract Broth for 21 days. Analyzed for secondary metabolites, the extract revealed the presence of phenolics, alkaloids, flavonoids, steroids and terpenoids. Further, Gas Chromatography Mass Spectroscopy (GCMS) analysis of the extract revealed the presence of several compounds including 3-nitropropanoic acid, 4H-pyran-4-one 5-hydroxy-2-(hydroxymethyl), hexadecanoic acid, and octadecanoic acid, known to be cytotoxic or antioxidative. Among different cell lines tested, HeLa cells were the most vulnerable to the treatment of the fungal extract with an IC_50_ value of 101 ± 1 μg/mL. The extract showed no significant cytotoxicity to the normal human embryonic kidney cell line (HEK 293 T) in the MTT assay. The ethyl acetate extract induced membrane damage and mitochondrial depolarization and thereby apoptosis and cytotoxicity in HeLa cells. The study marks marine-derived endophytes as potential sources for discovery of novel drugs.

## Introduction

Natural product research has occupied a prominent position in pharmaceutical industries and agriculture for development of high-value products for use in human healthcare, nutrition and therapeutics (Kusari et al., 2014; Gupta and Tuohy, 2015; Gill et al., 2016; Fei Law et al., 2017; Masand et al., 2018). Natural product chemistry has also played a vital role in providing better substitutes for existing drugs (Kusari et al., 2014; Kosanić et al., 2016), especially in dreaded diseases like cancer, a major cause of morbidity and mortality in developing and developed countries alike (Mallath et al., 2014; Kilcullen et al., 2016). Taxol® is a classic example of natural complex diterpenoids which has gained the status of a blockbuster anticancer drug. *Smallanthus sonchifolius* extracts induced G2/M arrest and apoptosis through mitochondria pathway in HeLa cell lines (Kitai et al., 2017) whereas *Rhus coriaria* extract (RCE) induced autophagy in MCF-7 cell lines by activating p38 and ERK1/2 (Hussain et al., 2015). Similarly, reactive oxygen species are implicated in many diseases including cancer (Tu et al., 2016; Hsieh et al., 2018). There is a constant urge to search for new, alternative bioactive compounds with anticancer and antioxidative activities. In agriculture also, endophytes are known to enhance plant productivity and stress tolerance by modulating plant molecular responses. In a recent study, Bilal et al. (2018) demonstrated plant growth promoting endophytic fungi *Asprgillus fumigatus* TS1 and *Fusarium proliferatum* BRL1 produced gibberellins and regulated plant endogenous hormones.

The oceans and seas comprise the most diverse and huge platforms for biodiversity. The complexity and extreme conditions marine organisms face in their niche for survival provides them with a unique potential to produce a large range of compounds (Hamed et al., 2015). Marine-derived endophytic fungi which colonize internal tissues of their host such as seaweeds, sponges, mangroves have emerged as alternative sources of promising bioactive agents, including alkaloids, terpenoids, polyketides, lipids, proteins, glycosides, isoprenoids, and hybrids of those metabolites (Debbab et al., 2011; Kjer et al., 2011). Compounds isolated from the culture extracts of *Talaromyces* and *Penicillium* sp. residing in marine sources have shown their potential as anticancer, antioxidative and antimicrobial agents (Debbab et al., 2011; Liu et al., 2016; Nicoletti and Trincone, 2016). Though many important secondary metabolites have been obtained from endophytic fungi, increasing their yield to fulfill the demand has always been a problem. Physico-chemical parameters play an utmost role in deciding the quantity of bioactive agent produced by endophytic fungi. For example, Valayil et al. (2015) showed the role of culture media, time and temperature for enhanced production of cholestanol glucoside and Venugopalan and Srivastava, 2015) elucidated the effect of fermentation parameters, elicitors and precursors on camptothecin production from the endophytic fungus *Fusarium solani*.

In the present study, we report antiproliferative and antioxidative activities of the culture extract of *Talaromyces purpureogenus*, an endophytic fungus isolated from a marine brown algal species, and possible mechanism of these effects.

## Materials and methods

### Chemicals and solvents

Dulbecco's Modified Eagle's Medium (DMEM), dimethyl sulphoxide (DMSO) were purchased from Sigma Aldrich, USA. Fetal Bovine Serum (FBS) and trypsin-EDTA were purchased from GIBCO-BRL. Growth media for fungi including Potato Dextrose Agar and Broth (PDA and PDB) were purchased from Himedia, India. Antibiotics and MTT reagent was procured from SRL-Ranbaxy. Ethyl acetate, hexane, chloroform, dichloromethane, methanol, diethyl ether and petroleum ether were purchased from Merck, India. All other chemicals and reagents were of analytical grade.

### Isolation of endophytic fungi from a seaweed and identification of an anticancer secondary metabolite-producing fungus

Endophytic fungi were isolated from brown algae collected from coasts at Kovalam, Trivandrum city, Kerala state, India (8.4004° N, 76.9787° E) following the protocol of Kjer et al. (2011). The fungal mycelia that emerged from the thallus fragments after a few days were transferred on the fresh PDA medium and incubated at 28 ± 2°C for 7 days to obtain pure cultures.

Out of five endophytic fungal isolates, only one was selected for the present study because of its relatively high antiproliferative activity in MTT assay of its extract on HeLa cells during the preliminary screening of culture extracts of isolated fungi. The fungus was identified as *T. purpureogenus* by sequencing of the rDNA ITS (internal transcribed spacer) region ITS1 (sequence: 5′-TCC GTA GGT GAA CCT GCG G-3′) and ITS4 (5′-TCC TCC GCT TAT TGA TAT GC-3′) (Kjer et al., 2011; Kuriakose et al., 2014).

### The growth of *T. purpureogenus* and preparation of fungal culture extracts

To study the effect of incubation time of *T. purpureogenus*, the total culture was harvested at different time intervals (7, 14, 21, 28, and 35 days) after inoculation in PDB. Further, the fungus in a 1 L flask containing 100 mL of nine different media viz. Gauce Medium (GA1), Malt Extract Broth (MEB), Potato Dextrose Broth (PDB), Sabourard Broth (SDB), Yeast Malt Extract Broth (YME), Yeast Extract Phosphate Broth (YEP), Czapek Yeast Extract Broth (CZB), Goose and Tschessch Broth (GTB), and Leonine Broth (LEB) (for chemical composition, see Table S1) was grown for 28 days in dark and static conditions, at 28°C. Two agar containing mycelium (0.5 × 0.5 cm) were used as inocula. After 28 days, the mycelia were separated out from the supernatant and crushed in liquid N_2_. Crushed mycelia were added with supernatant and extracted twice with equal volume of different solvents viz. hexane, petroleum ether, diethyl ether, chloroform, dichloromethane and ethyl acetate. Further, salinity (0, 1.5, and 3% NaCl) was also optimized in the selected growth medium. The solvents were evaporated at 40°C in the rotary evaporator to obtain concentrated extracts, stored in a refrigerator till further use.

### Determination of mycelia dry weight

At the end of the incubation period, the content of each culture was filtered using Buchner funnel through pre-weighed Whatman no. 3 filter paper and washed thrice with deionised water. The filters were dried overnight at 60°C in an oven and weighed.

### Secondary metabolite analysis

The preliminary screening of *T. purpureogenus* ethyl acetate (TPEE) crude extract was done for the presence of different secondary metabolites. Alkaloids, phenolics, flavonoids, peptides, and terpenoids were estimated by the Dragendroff's method, Folin-Ciocalteu method, AlCl_3_ method, Biuret method, and Salkowski method, respectively (Ashraf et al., 2015; Kalidindi et al., 2015). For determination of steroids, 1 mL of extract was mixed with 10 mL of chloroform and an equal volume of conc. H_2_SO_4_ added along the sides of the test tube and the green fluorescence formed was measured with yellow color in H_2_SO_4_ indicated the presence of steroids (Kalidindi et al., 2015).

### Analysis of volatile metabolites by GCMS

To investigate the compounds present in ethyl acetate (TPEE) extract of *T. purpureogenus*, GCMS analysis was carried out in an Agilent GCMS apparatus (GC: 7890A; MSD5975C) with a silica HP-5 capillary column (30 m−0.25 mm, ID, film thickness of 0.25 mm) coupled directly with single quadrupole MS. The chromatographic separations of metabolites were carried out following the protocol of Kavitha and Savithri (2017) and the peaks detected in GC were assigned as particular compounds through mass spectral data analysis software and NISTMS library data, 2008.

### Assessment of cytotoxicity by MTT assay

Cytotoxic effect of fungal extracts was determined by 3-(4, 5-dimethylthiazole-2yl)-2, 5-diphenyl tetrazolium bromide (MTT) assay (Kuriakose et al., 2014) in different human tumor cell lines viz. HeLa (cervical cancer), MCF-7 (breast cancer), Hep G2 (liver cancer), A549 (lung cancer), A-431 (skin cancer) and LN-229 (glioblastoma). Cytotoxicity of fungal crude extracts was also tested on normal human embryonic kidney cell line (HEK 293 T). Cells were cultured in DMEM medium supplemented with 10% FBS, 100 mg/L penicillin, 250 mg/L streptomycin and 2 mM glutamine and incubated at 37°C in a humidified chamber with 5% CO_2_.

Briefly, cells at a density of 1 × 10^4^ cells/mL were plated with 100 μL DMEM in 96 well plates and grown for 24 h at 37°C in a CO_2_ incubator. Thereafter, different concentrations of fungal extracts, ranging from 10 to 200 μg/mL were added into the respectively labeled wells. After 48 h of incubation, 10 μL of 0.5 mg/mL MTT was added in each well and incubated for 2 h at 37°C. Thereafter, the medium solution in PBS was removed and DMSO (100 μL) was added to each well and the absorbance was then determined by an ELISA reader (Molecular Devices, USA) at a wavelength of 595 nm. The relative growth inhibition in percentage was calculated by comparing the viability of the treated cells with that of control.

### Live dead cell assay using PI staining

TPEE mediated cell death in HeLa cells were studied using propidium iodide (PI) as per the protocol of Kumari et al. (2017) with minor modifications. The cells (1 × 10^4^/mL) were seeded in 24-well culture plate and exposed to different concentrations of TPEE (10, 50, 100, μg/mL) for 48 h. After the indicated incubation time, the cells were trypsinized and washed with PBS twice. PI (1 μg/mL) was added to each sample just before the acquisition of data in FACS Verse (Becton Dickinson, USA). Untreated cells served as control. The acquired data were analyzed using BD FACS DIVA™ software.

### Determination of nuclear morphology

Apoptotic nuclear morphological changes of HeLa cells after treatments with TPEE were observed after dual staining with acridine orange/propidium iodide (AO/PI). HeLa cells (1 × 10^4^ cells/well) were seeded in six-well plates on 0.01% poly-L-Lysine coated cover slips (24 mm) and were treated with different concentrations (10, 50, and 100 μg/mL) of TPEE for 48 h. Following incubation for 48 h, cells were washed with phosphate buffered saline (PBS) twice and stained with AO/PI (1 mg/mL) mixture for 2–3 min. The cells were then examined for apoptotic cell death under confocal microscope (Epi-fluorescence Olympus DSU, Japan) (Zahedifard et al., 2015).

### Mitochondrial membrane potential assessment

The mitochondrial membrane potential (MMP or ΔΨm) was measured using the potentiometric dye JC-1 as described by Kuriakose et al. (2014). Briefly, HeLa cells were incubated at 1 × 10^4^ cells per well in 24 well plate with different concentrations of TPEE (10, 50, and 100 μg/mL) for 24 h in 10% FBS in DMEM medium. After incubation, cells were stained with 2.5 μg/mL of JC-1 dye at 37°C for 15 min in the CO_2_ incubator in dark conditions. Further, cells were trypsinized and washed twice with PBS and analyzed immediately in flow cytometry using FACS Verse (Becton Dickinson, USA) at an excitation/emission wavelength of 488 nm and 530/30, 585/42 nm, respectively and analyzed by BD FACS DIVA™ software. The emission of JC-1 monomers peaks at 530 nm (FL-1 channel- green fluorescence) while that of J aggregates peaks at 590 nm (FL-2 channel-red fluorescence). 2,4-Dinitrophenol (2,4-DNP) treated cells served as the positive control and untreated cells served as the negative control.

### Measurement of intracellular reactive oxygen species (ROS)

The involvement of ROS production in TPEE mediated apoptosis was studied using the specific ROS sensitive fluorescent probe 2′,7′-dichlorodihydrofluorescein diacetate (DCFH-DA), (Sigma Aldrich, USA) (Kumari et al., 2017). Briefly, HeLa cells (1 × 10^4^ cells) were seeded in 24 well plates, incubated for 24 h and treated with different concentrations (10, 50, and 100 μg/mL) of TPEE for 24 h. Further, cells were washed with PBS and stained with 50 μM of DCFH-DA for 15 min at 37°C in dark. H_2_O_2_ (800 μM) served as positive control and untreated cells served as negative control. Fluorescence generated due to oxidation of DCFH was measured by flow cytometry at 500 nm using BD FACS Verse (Becton Dickinson, USA) and analyzed by BD FACSUITE Software.

### DPPH scavenging activity

Antioxidative activity of the extracts was determined by the 1,1-diphenyl-2-picrylhydrazyl (DPPH) radical scavenging activity (Kalidindi et al., 2015; Ser et al., 2015). Briefly, the extract (1 mL of 10 and 50 μg/mL extracts) was added to 1 mL of 100 μM DPPH in methanol. The mixture was vortexed for 1 min and left in the dark for 30 min at room temperature. Absorbance values were measured at 517 nm using an ELISA reader against methanol as a blank (Molecular Devices, USA). The scavenging activity of DPPH radicals was calculated by assessing decrease in purple color of DPPH. Ascorbic acid served as positive control and DPPH without the addition of extract served as negative control.

### Statistical analysis

All the experiments were performed in triplicate and quantitative variables are represented in terms of mean ± S.D. in histograms. For statistical significance, means ± SD of all groups were compared and analysis of variance (ANOVA) was performed using a statistical package, SPSS 16.0 (SPSS Inc., Chicago, IL, USA). A probability of *P* ≤ 0.05 was taken to indicate statistical significance. Further, Duncan's Multiple Range Test (DMRT) was used to identify the pairs of groups where the means are significantly different at α = 0.05.

## Results

### Isolation and identification of endophytic fungus

The screened fungus was identified as *Talaromyces purpureogenus* using ITS rDNA sequencing. The partial identified sequence has been submitted to Genbank with accession number MG807065.

### Standardization of culture conditions and organic solvents for growth and antiproliferative activities of different extracts of cultures of *T. purpureogenus*

**Figure 1:**
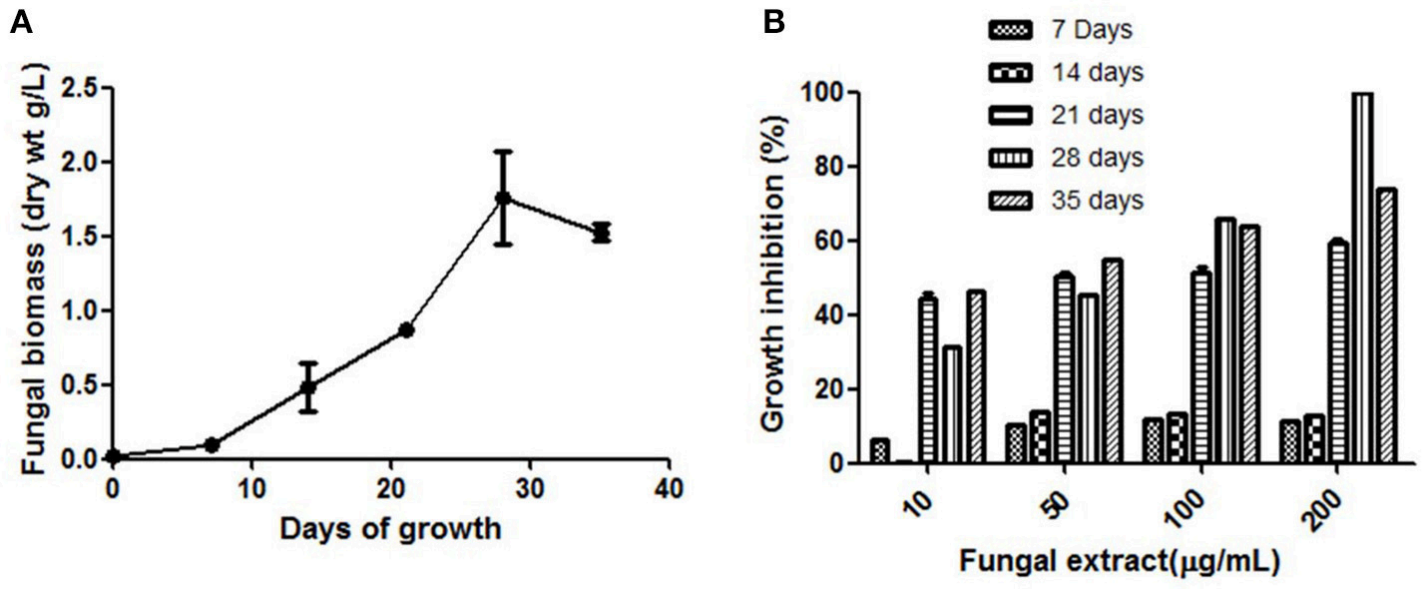
**(A)** Time course of biomass production by *T. purpureogenus* in PDB culture medium. **(B)** Time course of cytotoxic secondary metabolites production by *T. purpureogenus*. The fungus was grown in PDB for indicated time period and the ethyl acetate extracts of the fungal culture were tested for cytotoxic activity against HeLa cell line by MTT assay. Values are the means ± SD of three independent experiments.

**Figure 2:**
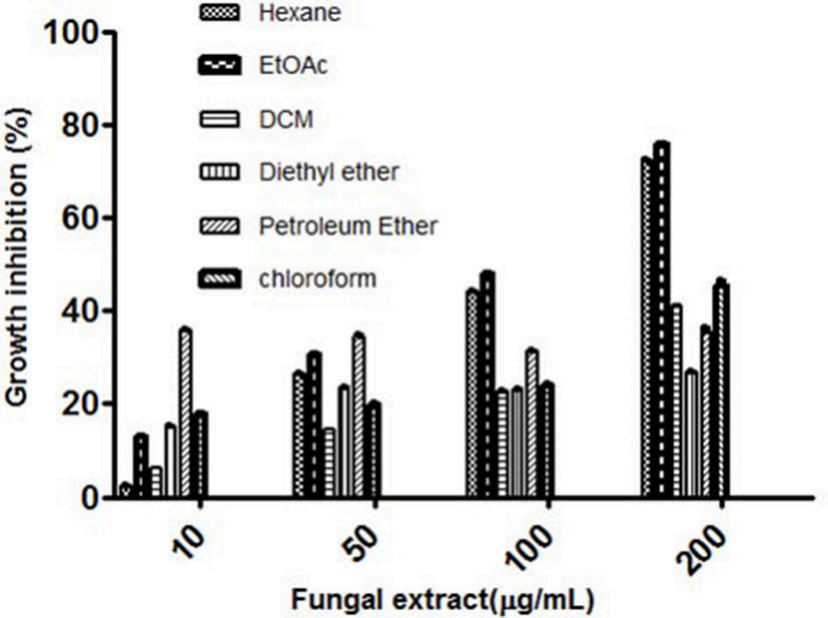
Cytotoxic effects of different solvent extracts of *T. purpureogenus* against HeLa cell line by MTT assay. HeLa cells were treated with indicated concentration of different solvent extracts for 48 h. Values are the means ± SD of three independent experiments.

The fungal growth was observed up to 35 days and the highest mycelial biomass and highest cytotoxic activity were observed on day 28 (Figures 1A,B). The solvent extraction method was used to extract the cytotoxic secondary metabolites with several organic polar and non-polar solvents. We initially investigated the effect of different organic extracts of total culture on the proliferation of HeLa cells. All the extracts showed some growth inhibitory effect against HeLa cells but ethyl acetate fraction (TPEE) showed strong inhibitory effects (Figure 2). For further work based on this result ethyl acetate extract was selected.

### Optimization of culture media for growth and cytotoxic secondary metabolites production by *T. purpureogenus*

**Figure 3:**
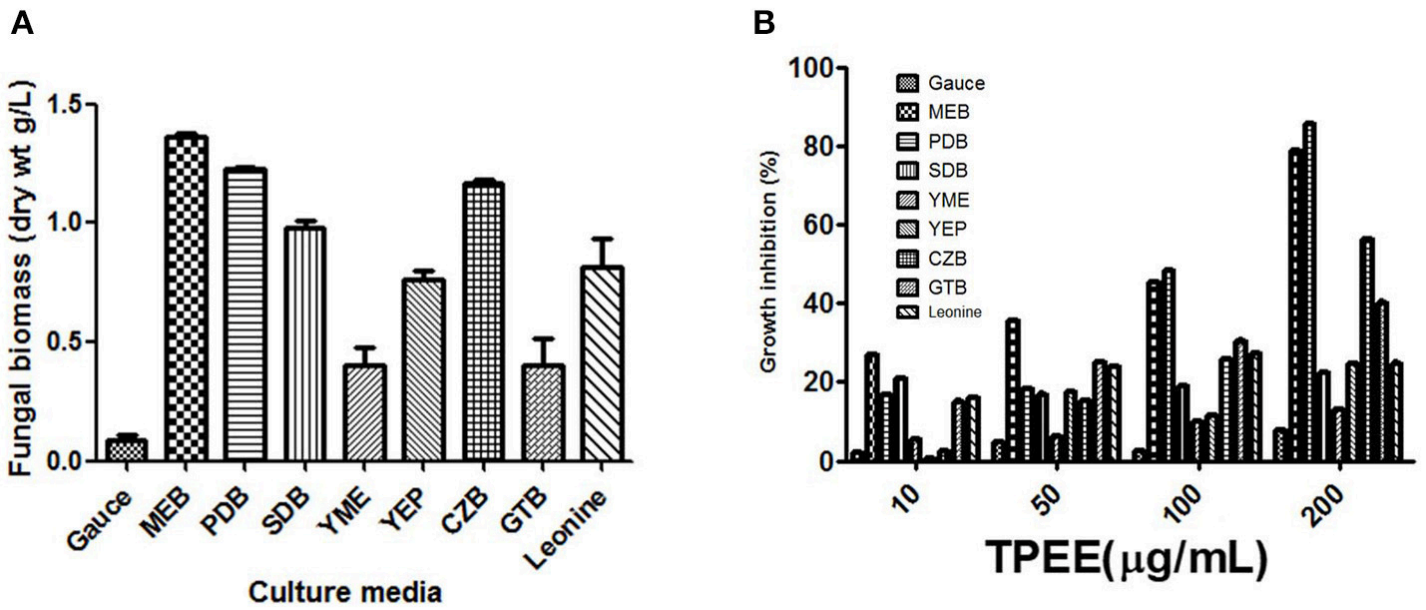
**(A)** Production of biomass in different culture media. The fungus was grown in different media for 28 days and the biomass was harvested as described in methods. **(B)** Effect of different culture media on the production of cytotoxic secondary metabolites by *T. purpureogenus*. The fungus was grown in different media for 28 days and the ethyl acetate extracts (TPEE) were tested for cytotoxic activity. Values are the means ± SD of three independent experiments.

**Figure 4:**
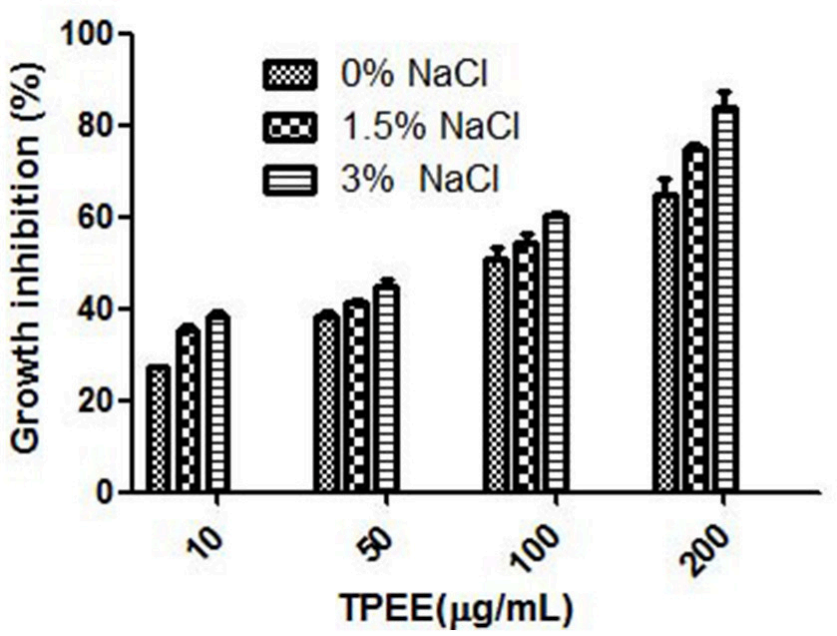
Effect of NaCl on the production of cytotoxic secondary metabolites by *T. purpureogenus*. The fungus was grown in PDB with different concentration of NaCl for 28 days and the ethyl acetate extracts (TPEE) were tested for cytotoxic activity. Values are the means ± SD of three replicates.

Nine different growth media were tried at shake flask level to find the appropriate medium for the optimum growth of the organism and anticancer cytotoxic secondary metabolite production. The production of biomass from different media varied between 0.3 ± 0.002 and 1.36 ± 0.1 g/L (Figure 3A). The maximum biomass was obtained from MEB medium following PDB and CZB. The extract of same nine different liquid culture media when treated for their cytotoxicity (due to production of cytotoxic secondary metabolites) against the HeLa cell lines showed discrete cytotoxicity pattern (Figure 3B). The results obtained by MTT assay showed the highest cytotoxic activity was obtained from growing it in PDB and MEB media with the IC_50_ values of 103 ± 2.4 and 109 ± 3.1 μg/mL, respectively. Further, among three different concentrations of salinity tested for antiproliferative activity of the extract, a concentration-dependent increase was observed in extract obtained from PDB with 3% supplementation of NaCl, mimicking natural seawater (Figure 4).

### Presence of different secondary metabolites in the fungal extract (TPEE)

After physico-chemical optimization, the fungal extract was examined for presence of different groups of secondary metabolites. It was found to have phenolics, alkaloids, flavanoids, steroids and terpenoids as major groups of secondary metabolites in fungal crude extract.

### Analysis of volatile compounds by GCMS

Gas Chromatography Mass Spectroscopy (GCMS) analysis indicated that 61 compounds are present in TPEE (Table 1). Occurrence in fungi of 3-nitropropanoic acid having anti-micobacterial and cytotoxic properties has earlier been reported by Chomcheon et al. (2005) and Hollmann et al. (2008). Propanoic acid acts as a precursor of siderophore and several antibiotic agents (Kumari et al., 2017). Ethanol and ethanone present in relatively high concentration in TPEE might also contribute toward the cytotoxicity. 4H-pyran-4-one, 5-hydroxy-2-(hydroxymethyl), commonly known as Kojic acid, is a potent antioxidative agent (Frisvad, 2014) but its cytotoxic activity is yet unexplored. Recently, cytotoxicity of hexadecanoic acid extracted from *Kigelia pinnata* leaves against human colorectal carcinoma has been demonstrated by interaction with DNA topoisomerase I (Ravi and Krishnan, 2017). Hexadecanoic acid and octadecanoic acid are also known for their potent antioxidative activities (Patra et al., 2015). Dibutyl phthalate has also shown cytotoxicity by inducing apoptosis (Wo'jtowicz et al., 2017).

**Table 1:** Quantification of metabolites present in ethyl acetate crude extract (TPEE) of *T. purpureogenus* by GCMS analysis.

**S.no**	**RT (min)**	**Metabolites**	**Relative concentration (%)**
1.	1.518	Urea	2.948688 ± 0.1
2.	1.578	3-Nitropropanoic acid	1.323096 ± 0.1
3.	1.668	Propanoic acid	1.671578 ± 0.03
4.	1.743	Ethanol, 2-nitro-, propionate (ester)	1.590 ± 0.01
5.	2.12	2-Pentene, 4-methyl-, (E)-	0.543632 ± 0.003
6.	2.315	Butanamide, 3-methyl-	0.556617 ± 0.002
7.	2.722	1H-Pyrrole, 2-methyl-	0.4712 ± 0.002
8.	2.993	Pyridine, 2,6-dimethyl	0.11371 ± 0.004
9.	3.324	Dimethyl sulfone	0.099665 ± 0.003
10.	3.73	1-Butanol, 4-methoxy-	0.208109 ± 0.008
11.	6.243	3-Heptanone	0.17252 ± 0.005
12.	7.688	4-Methyl-2-oxo-(1H)-pyrimidine	0.398271 ± 0.001
13.	8.425	2-Hydroxy-5,5-dimethyl-hex-2-en-4-one	0.24555 ± 0.004
14.	10.095	1H-Pyrazole, 4,5-dihydro-5,5-dimethyl-4-isopropylidene-	0.068606 ± 0.06
15.	10.366	1H-Pyrrole-2-carboxaldehyde, 1-methyl-	0.209602 ± 0.05
16.	14.791	Ethanone, 1-(1-methyl-1H-pyrrol-2-yl)-	3.263856 ± 0.01
17.	15.588	Ethanol, 2-(2-butoxyethoxy)-	2.517 ± 0.01
18.	21.954	Ethanol, 2-(2-butoxyethoxy)-, acetate	0.517581 ± 0.01
19.	22.992	1-Dodecene	0.8309 ± 0.01
20.	22.917	Phthalic acid, isobutyl 4-octyl ester	0.423226 ± 0.02
21.	23.73	Indolizine, 3,5-dimethyl-	0.356049 ± 0.008
22.	25.761	4H-Pyran-4-one, 5-hydroxy-2-(hydroxymethyl)-	1.080993 ± 0.02
23.	31.42	Phenol, 2,4-bis(1,1-dimethylethyl)-	0.395202 ± 0.008
24.	37.876	Dodecanoic acid	0.254661 ± 0.005
25.	39.876	2-Piperidin-1-yl-6,7-dihydro-oxazolo[3,2-a][1,3,5]triazin-4-one	0.152266 ± 0.001
26.	40.72	Diethyl Phthalate	0.109352 ± 0.004
27.	40.885	3-Hexadecene, (Z)-	2.621673 ± 0.06
28.	41.999	Hexadecane	0.202634 ± 0.05
29.	42.797	Pentadecane, 1-methoxy-13-methyl-	0.140488 ± 0.004
30.	44.00	Benzophenone	0.330142 ± 0.002
31.	44.407	Pentadecane, 1-methoxy-13-methyl-	0.215737 ± 0.004
32.	46.228	Benzaldehyde, 4-(diethylamino)-	0.133138 ± 0.002
33.	46.273	Indole-2-one, 2,3-dihydro-5-hydroxy-1,3-dimethyl-	0.231377 ± 0.004
34.	54.339	2H-1-Benzopyran-2-one, 3,5,7-trihydroxy-	1.684954 ± 0.03
35.	61.547	Phenol, 4-(1,1,3,3-tetramethylbutyl)-	5.506252 ± 0.01
36.	64.587	2-(1-Hydroxyethyl) hydroxymethyl benzene	1.485532 ± 0.03
37.	66.062	Tricyclo [3.3.1.1(3,7)] decanone, 4-iodo-, (1α,3β,4α,5α,7β)-	1.994276 ± 0.04
38.	66.859	1,4-Benzenediol, 2,5-bis(1,1-dimethylethyl)-	0.244313 ± 0.005
39.	72.909	4′-Propoxy-2-methylpropiophenone	0.977758 ± 0.02
40.	77.213	Hexadecanoic acid, methyl ester	1.611505 ± 0.03
41.	86.243	Tetradecanoic acid	0.42833 ± 0.008
42.	94.007	E-15-Heptadecenal	5.25804 ± 0.1
43.	97.108	Dibutyl phthalate	11.30445 ± 0.23
44.	100.208	4a, trans-8a-Perhydro-cis-2-(2-hydroxy-2-propyl)-4a,cis-8-dimethylnaphthalene	2.342847 ± 0.004
45.	126.408	1,2-Benzenedicarboxylic acid, bis(2-methylpropyl) ester	0.285096 ± 0.005
46.	138.311	9-Eicosene, (E)-	0.46629 ± 0.009
47.	143.274	2-Methyl-7-nonadecene	0.313766 ± 0.006
48.	147.05	Vinylbital	0.08709 ± 0.001
49.	155.256	n-Hexadecanoic acid	16.37683 ± 0.339
50.	156.008	Behenic alcohol	9.531588 ± 0.19
51.	156.881	7,9-Di-tert-butyl-1-oxaspiro (4,5) deca-6,9-diene-2,8-dione	1.14348 ± 0.02
52.	157.649	Phthalic acid, butyl ester, ester with butyl glycolate	0.0797 ± 0.001
53.	158.582	Hexadecanoic acid, ethyl ester	0.101213 ± 0.002
54.	161.787	Eicosane	1.168244 ± 0.02
55.	168.574	2-Methyl-7-nonadecene	0.343921 ± 0.007
56.	168.584	3-Eicosene, (E)-	0.76107 ± 0.01
57.	169.573	Benzenepropanoic acid, 3,5-bis(1,1-dimethylethyl)-4-hydroxy-, methyl ester	0.110915 ± 0.002
58.	180.237	Hexadecanoic acid, methyl ester	0.079235 ± 0.02
59.	198.175	Dibutyl phthalate	9.696606 ± 0.08
60.	212.562	Octadecanoic acid	4.1082 ± 0.08
61.	213.434	1-Docosene	0.54764 ± 0.01

*Values are the means ± SD of three independent experiments*.

Hexadecane, 2H-1-Benzopyran-2-one, 3,5,7-trihydroxy, phenol, 4-(1,1,3,3-tetramethylbutyl), 2-(1-Hydroxyethyl)hydroxymethylbenzene,Tricyclo[3.3.1.1(3,7)] decanone, trans-8a-Perhydro-cis-2-(2-hydroxy-2-propyl)-4a,cis-8-dimethylnaphthalene, Heptadecenal, Behenic alcohol, vinylbital and eicosane are some of the other major compounds found in TPEE whose biological potential is yet to be explored.

### The cytotoxic effect of TPEE in various cancer cell lines

**Figure 5:**
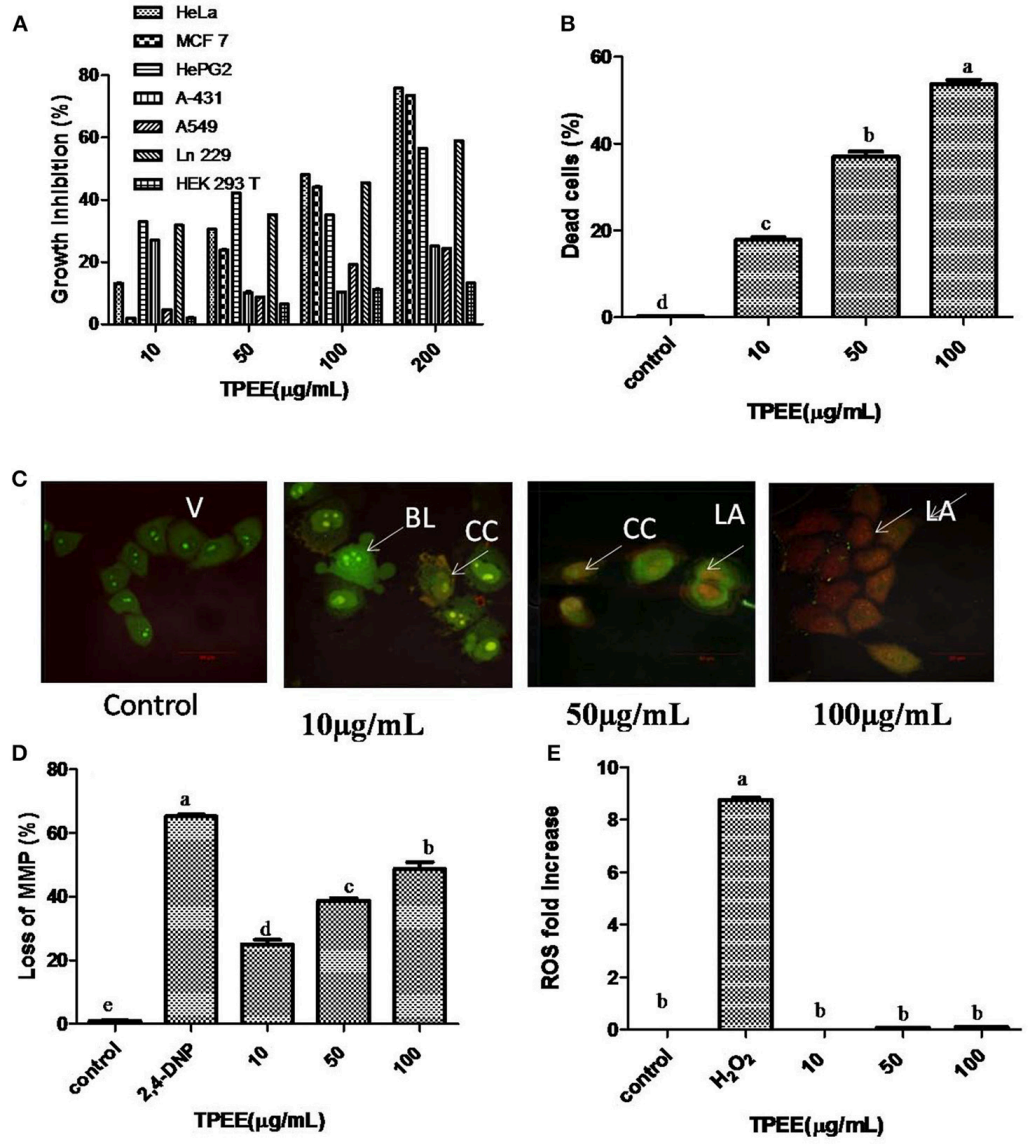
Inhibition of cell growth and induction of apoptotic cell death by the fungal extract. **(A)** Inhibition of cell growth by the TPEE on various cancer cell lines. Cells were treated with indicated concentrations of the extract for 48 h and the inhibitory effects of the extract on cell growth was determined by assessing the cell viability using MTT assay. **(B)** Fungal extract induced cell death in HeLa cells. Hela cells were treated with the indicated concentrations of TPEE for 48 h, stained with PI and subjected to flow cytometry analysis to evaluate live and dead population. **(C)** Induction of apoptotic nuclear morphology by TPEE. HeLa cells were treated with the indicated concentrations of the extract and stained with AO/PI dual staining. V, viable cells; CC, chromatin condensation; BL, membrane blebbing; LA, late apoptotic cells. **(D)** Induction of loss of mitochondrial membrane potential by the extract. Cells were treated with the indicated concentrations of TPEE for 48 h and subsequently analyzed for change in mitochondrial membrane potential by JC-1 using flow cytometry. 4-DNP treated cells served as positive control. **(E)** Induction of ROS potential by TPEE. Cells were treated with indicated concentrations of the extract for 24 h and stained with DCFH-DA and intracellular ROS was monitored by flow cytometry. Values are the means ± SD of three replicates. Means sharing different alphabets “a,” “b” differ significantly from each other at *p* ≤ 0.05.

The cells were treated with various concentrations of TPEE ranging from 10 to 200 μg/mL to obtain IC_50_ values, shown in Figure 5A. TPEE displayed moderate growth inhibitory effects toward all human cell lines tested in a concentration-dependent manner. More significantly, TPEE displayed good cytotoxicity toward HeLa and MCF-7 cell lines with IC_50_ values of 101 ± 1.4 and 110 ± 3 μg/mL respectively. The cytotoxic effect of TPEE on human embryonic kidney cell line, HEK 293T was turned to be negligible, proving TPEE is non-toxic to the non-cancerous cell. We decided to use HeLa cells for further investigation for part of this study.

### Induction of apoptosis by TPEE in HeLa cells

We further investigated the possible mechanism of the inhibitory effect on HeLa cells for TPEE. In order to confirm the cytotoxic effects of TPEE on the proliferation of HeLa cells, live-dead cell assay was performed. The data showed that treatment of cells with TPEE reduced the viability of HeLa cells in a concentration-dependent manner (Figure 5B, Figure S1). Consistent with above results, cell viability was affected 55.2% cell death at 100 μg/mL of TPEE.

We further used AO/PI double staining to determine the effect of TPEE on HeLa cell morphology. Most of the cells in untreated control had green nuclei (Figure 5C) whereas HeLa cells treated with increasing concentrations of TPEE showed a predominant red nuclei staining due to PI uptake and were characterized by shrinkage in cell volume and membrane blebbing as well, as indicated in the early stage of apoptosis. Typical apoptotic features such as chromatin condensation, deformed and fragmented nuclei associated with late apoptosis were also observed.

ΔΨm was monitored by flow cytometry using JC-1 dye. As shown in Figure 5D, TPEE induced a concentration-dependent increase in the proportion of HeLa cells with depolarized mitochondria compared with 0.8% in the control cells. The number of cells with loss of MMP increased to 22.4, 38.2, and 46.4%, respectively after the treatment with 10, 50, and 100 μg/mL of TPEE after 24 h (Figure 5D; Figure S2).

Mitochondrial membrane depolarization is also associated with the production of reactive oxygen species (ROS). To investigate whether TPEE stimulates ROS production in HeLa cells, we measured intracellular ROS by flow cytometry method following staining with DCFH-DA method. TPEE did not induce the production of ROS (Figure 5E, Figure S3).

### Standardization of culture conditions, organic solvents, culture media and salinity for antioxidative activities of different extracts of cultures of *T. purpureogenus*

**Figure 6:**
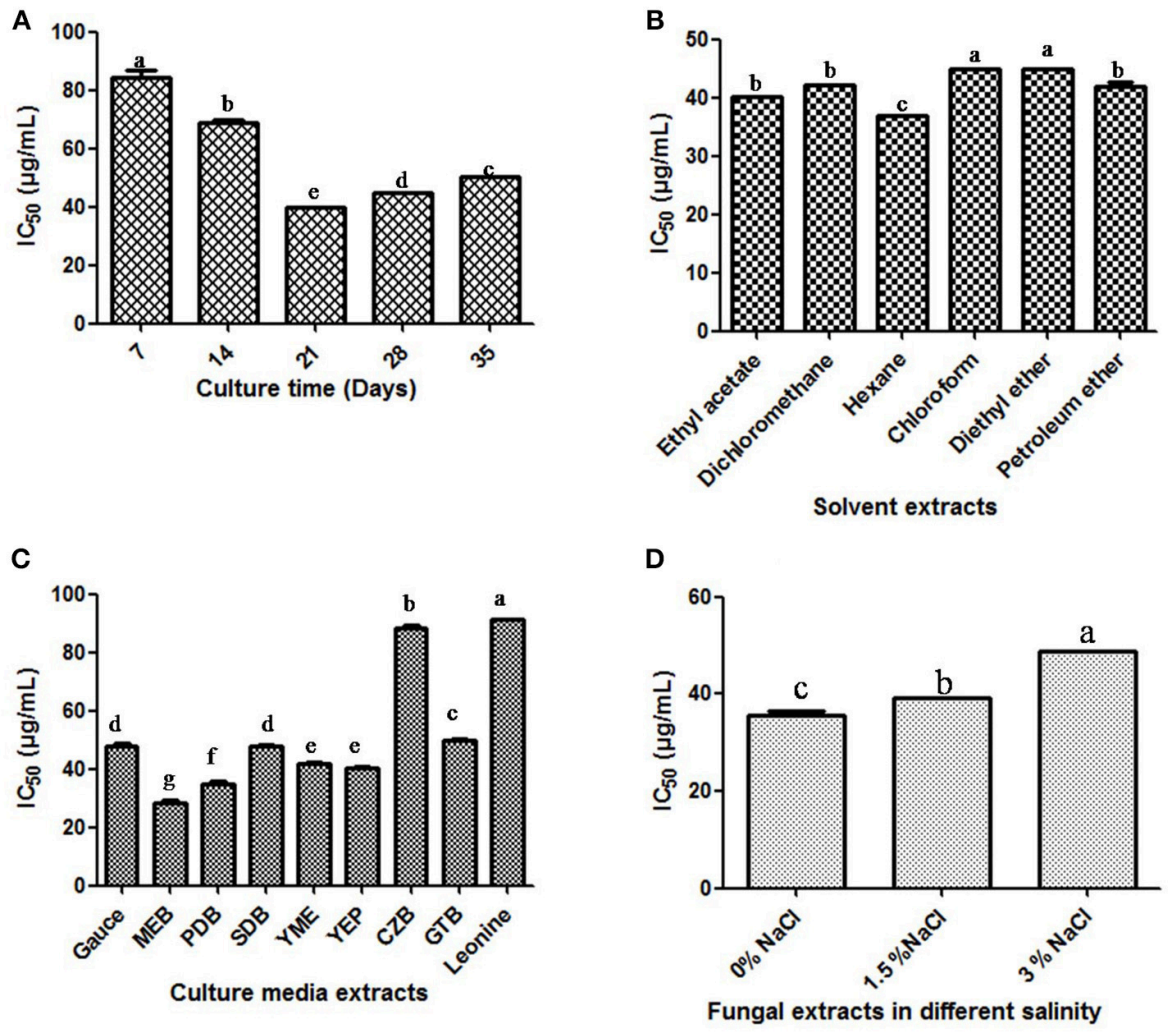
Production of antioxidant secondary metabolites by *T. purpureogenus*. The fungus was grown in various culture media for different time period and organic extracts of the total fungal culture were tested for their potential antioxidative activity (IC_50_ values) by DPPH radical scavenging assay. **(A)** Time course of DPPH scavenging of fungal crude extract of *T. purpureogenus*. The fungus was grown in PDB for indicated time period and the ethyl acetate extract was tested for antioxidative activity. **(B)** *In vitro* DPPH scavenging activity of different solvent extracts of *T. purpureogenus*. The fungus was grown in PDB for 21 days and different solvent extracts were tested for antioxidative activity. **(C)** Effect of different culture media on the production of antioxidative secondary metabolites by *T. purpureogenus*. The fungus was grown in different media for 21 days and the hexane extracts (TPHE) were tested for antioxidative activity. **(D)** Effect of NaCl on the production of antioxidant secondary metabolites by *T. purpureogenus*. The fungus was grown in MEB with different concentration of NaCl for 21 days and the hexane extracts (TPHE) were tested for antioxidative activity. Values are the means of three replicates ± SD of three replicates. Means sharing different alphabets “a,” “b” differ significantly from each other at *p* ≤ 0.05.

In order to optimize the culture conditions for *in vitro* DPPH scavenging activity of different extracts of cultures of *T. purpureogenus*, shake flask experiments were carried out at different culture parameters (Figure S4). The maximum potential of *in vitro* DPPH scavenging in total culture extract was observed after 21 days of inoculation (Figure 6A). Different non-polar, semi-polar and polar organic solvents were used for the maximum extraction of antioxidative secondary metabolites. Among all solvents, hexane extract of total culture of *T. purpureogenus* showed maximum *in vitro* DPPH scavenging potential (Figure 6B). Among the nine media tested, MEB emerged as the best medium for production of antioxidative secondary metabolites with an IC_50_ value of 11.23 ± 0.012 μg/mL (Figure 6C). Further, salinity was also optimized in MEB media for maximum production of antioxidative secondary metabolites. Media without any supplementation of NaCl showed highest *in vitro* DPPH scavenging activity (Figure 6D). Ascorbic acid served as the positive control with an IC_50_ value of 28.39 ± 0.4 μg/mL.

## Discussion

Marine organisms are the cradle of birth for many excellent pharmaceutical products (Blunt et al., 2017; Shirley et al., 2018). Endophytes dwelling inside the marine seaweeds is the treasure house of many medicinally important secondary metabolites. The exceptional micro-environment faced by them to live as an endosymbiont in a marine surrounding seems to make them evolve distinct metabolites.

Endophytes have established their pivotal roles in both medicine and agriculture. Endophytic fungi living as close symbiont with plants play an important role in plant growth promotion, increasing vigor and plants resistant toward biotic and abiotic stresses (Rai et al., 2014). Endophytic fungus *Piriformospora indica* has received a great attention in agriculture by promoting plant growth and ameliorating plant stress (Varma et al., 2012; Gill et al., 2016). Further, recent studies of Mathur et al. (2018), showed improved photosynthetic efficacy of maize (*Zea mays*) plants with arbuscular mycorrhizal fungi (AMF) under high temperature stress. The secondary metabolites present in plant and endophytic extracts often act as elicitor or signal molecules to ameliorate biotic or abiotic plant stress (Sytar et al., 2018). In medicine and pharmaceutical industries, as well, secondary metabolites present in plant and endophytes have contributed toward the developments of novel drugs and medicine (Konaté et al., 2013; Li et al., 2017).

In this study, the endophytic fungus was identified as *T. purpureogenus. Talaromyces sp*. of marine origin is known for synthesis of several bioactive compounds (Nicoletti and Trincone, 2016). Earlier, metabolites obtained from *T. purpureogenus* have shown antibacterial and antifungal properties (Li et al., 2017). The qualitative chemical screening of crude extract showed the presence of phenolics, alkaloids, flavanoids, steroids and terpenoids as major secondary metabolite constituent. Presence of such a variety of compounds in the fungus makes it a potential candidate for exploration of bioactive compounds (Hulikere et al., 2016). Further, GCMS analysis revealed presence in TPEE of many cytotoxic and antioxidative compounds including 3-nitropropanoic acid, 4H-pyran-4-one, 5-hydroxy-2-(hydroxymethyl), hexadecanoic acid, and octadecanoic acid. A bioassay-based purification needs to be followed to get the most potent active principle from the pool of metabolites (Senges et al., 2018).

The major hurdle faced during use of natural products in the pharmaceutical industry is their low yield. Many fermentation parameters including incubation time, solvent extraction and media conditions have been optimized for enhanced production of the secondary metabolite of interest (Venugopalan and Srivastava, 2015; Yuan et al., 2015; Tian et al., 2016; Venugopalan et al., 2016). In this study, maximum biomass was observed after 28 days of growth of the fungus and the antiproliferative activity was also high in the ethyl acetate extract (Figures 1, 2). The secondary metabolites are the metabolic intermediates, important for defense and coping with environmental stress. Useful for long term use, they are produced in late log and stationary phase of growth (Bourgaud et al., 2001). In this study also, fungus reached the stationary stage 28 days after inoculation which was also the time for the highest cytotoxic activity (Figure 1). The maximum biological activity of fungal crude was observed in ethyl acetate and hexane extracts demonstrating the non-polar and semi-polar nature of bioactive compounds. Similar organic solvent dependent *in vitro* antioxidant activity of *Leea macrophylla* and screening applications in zebrafish embryo have been reported by Joshi et al. (2016) and Maes et al. (2012).

OSMAC approach (One Stain Many Compounds) can be utilized to observe maximum yield of a variety of secondary metabolites by manipulating the culture conditions and media optimization (Wu et al., 2015; Hemphill et al., 2017). A change in culture media can completely be reflected in metabolite profiling of the endophytes. Among the nine different media tested in the study, maximum biomass and antioxidative activity were obtained with MEB while cytotoxic activity was found highest with endophyte grown in PDB (Figure 3). Because of difference in nitrogen and carbon sources, MEB yielded maximum biomass and showed high antioxidative activity (Wu et al., 2015). Wu et al. (2015) have also found higher cytotoxic activity endophytic fungi *Morinda citrifolia* in MEB broth. For growth and accumulation of secondary metabolites, in marine sources, salinity plays an important role (Arumugam et al., 2014). To cope up with the saline environment of sea and oceans, marine endophytes have evolved a distinct metabolism pattern from their terrestrial counterparts. The physiological responses of marine organisms differ significantly from the terrestrial endophytes in presence and absence of different salt concentrations (Jingjing et al., 2011). In this study, maximum cytotoxic activity was observed with supplementation of 3% NaCl, while antioxidative activity was highest without any supplementation of NaCl. Arumugam et al. (2014) demonstrated the maximum production of bioactive compounds from piezotolerant fungus *Nigrospora* sp. when supplemented with 7.5% salinity. This depicts the presence of several biological active compounds in one fungal strain.

To obtain maximum biological activity from extract of the endophytic fungi, optimization of physico-chemical parameters is essential (Joshi et al., 2016; Kumari et al., 2016). After the optimization, cytotoxic activity of the fungal extract was checked on different human tumor cell lines. Maximum cell death was obtained with human cervical cancer cell line HeLa (IC_50_-101 ± 1.4 μg/mL) and minimal cell death was observed with human lung cancer cell line A549 (IC_50_- >200 μg/mL). All tumor cell lines were susceptible to the drug prepared with crude extract indicating the broad spectrum anticancer activity of the fungal extract. The minimal cytotoxic activity on non-cancerous cell line (HEK 293 T) makes the fungal extract safe for isolation of anticancer compounds and for use in pharmaceutical industries (Figure 5A).

An in-depth study was also carried out to decipher the mechanistic aspect of cancer cell death. A concentration-dependent increase in PI positive cells indicating cell death were observed during flow cytometry in accordance with the MTT results obtained earlier (Figures 5A,B, Figure S1). Propidium iodide is a cell impermeable nucleic acid stain and internalized only when the membrane is compromised (Kumari et al., 2017). Further, no ROS generation by cancer cells during drug treatment was observed by DCFH-DA staining in flow cytometry. DCFH-DA is a ROS specific fluorescent probe which gets oxidized to DCF (dichlorofluorescein) in the presence of ROS (Kumari et al., 2017). Strong antioxidative nature of the fungal extract might have inhibited essential generation of ROS inside the HeLa cells. Thomas et al. (2016) also studied similar observation after treating cancer cells with a novel resveratrol based tubulin inhibitor. A concentration-dependent mitochondrial membrane depolarization was observed after drug treatment (Figure 5D, Figure S2) indicating induction of apoptosis by JC-1 staining with flow cytometry. The JC-1 is a cationic dye, fluoresces red when mitochondria are intact, but when the mitochondrial membrane potential is absent, it accumulates in the cytoplasm as monomers and emits green fluorescence. Mitochondrial depolarization is an early event of apoptosis leading to different molecular changes for apoptotic cell death (Thomas et al., 2016). To confirm apoptosis, AO/PI double staining was also carried out (Figure 5C). This assay is based on the emission of green and orange fluorescent wavelengths by AO and PI, respectively. A concentration-dependent increase in orange fluorescing cells indicating late apoptosis was observed after 48 h of drug treatment. Early apoptotic cells with a characteristic feature of membrane blebbing and chromatin condensation were also observed confirming the apoptotic cell death of cancer cells.

The excellent anticancer and antioxidative activity demonstrated by the extract of *T. purpureogenus* indicates the potential of marine endophytes as pharmaceutical source.

## Conclusion

The present study was carried out to explore the antiproliferative and antioxidative potential of endophytic fungus *T. purpureogenus* obtained from marine seaweed. After physico-chemical optimizations of growth conditions, media, salinity and solvent systems, maximum antiproliferative activity was observed in ethyl acetate extract (TPEE) of total culture grown in PDB for 28 days, while maximum antioxidative activity was found in hexane fungal extract grown in MEB for 21 days. Maximum cytotoxicity activity of TPEE was observed against HeLa followed by MCF-7 and HePG2 cell lines. The extract was able to induce apoptosis in HeLa cell line indicating programmed cell death of tumor cells as observed by mitochondrial depolarization and AO/PI staining. Isolation and purification of bioactive compounds from the isolate is underway to get novel compounds which could be exploited in the treatment of cancer.

## Author contributions

The work was conceived and designed by CJ. Experiments were done by MK, ST, and AS. Data analysis was done by MK. The manuscript was drafted by MK and CJ. The manuscript was approved by all the authors.

### Conflict of interest statement

The authors declare that the research was conducted in the absence of any commercial or financial relationships that could be construed as a potential conflict of interest.
