# Supplementary material for: Antiproliferative and Antioxidative Bioactive Compounds in Extracts of Marine-Derived Endophytic Fungus Talaromyces purpureogenus
Source: Front Microbiol. 2018 Aug 3;9:1777. doi: 10.3389/fmicb.2018.01777 (PMC6085570; doi:10.3389/fmicb.2018.01777)
Supplement: Supplementary file 1 [file Data_Sheet_1.docx]

**Supporting Information**

**Antiproliferative and Antioxidative Bioactive Compounds in Extracts of Marine-Derived Endophytic Fungus *Talaromyces purpureogenus***

**Madhuree Kumari, Sidhartha Taritla, Ankur Sharma, C Jayabaskaran***

*Department of Biochemistry, Indian Institute of Science, Bangalore-560012, India*

***Corresponding author:**

Prof. C. Jayabaskaran,

Department of Biochemistry,

Indian Institute of Science, Bangalore-560012,

India,

Tel: +91-80-22932482; Fax: +91-80-23600814;

E-mail: [cjb@iisc.ac.in](mailto:cjb@iisc.ac.in)

**Table S1: Different media used for the optimization of fungal anticancer secondary metabolites production**

| **Liquid Media** | **pH** | **Composition (g/L)** |
| --- | --- | --- |
| Gauce medium | 7.4 | Soluble starch 20; NaCl 0.5; KNO_3_ 1; K_2_HPO_4_.3H_2_O 0.5; MgSO_4_.7H_2_O 0.5; FeSO_4_.7H_2_O 0.01 |
| Malt Extract Broth (MEB) | 7.0 | Malt extract 20; Glucose 20; Peptone 1 |
| Potato Dextrose Broth (PDB) | 6.0 | Potato infusion 4; Dextrose 20 |
| Sabourard Broth (SDB) | 5.6 | Tryptone 10; Glucose 40 |
| Yeast Malt Extract Broth (YME) | 6.2 | Malt extract 10; yeast extract 4; MgSO_4_ 0.5; KH_2_PO_4_ 0.5 |
| Yeast Extract Phosphate Broth (YEP) | 6.0 | Yeast extract 1; KH_2_PO_4_ 6; NaH_2_PO_4_ 4; NH_4_OH 1 |
| Czapek Yeast Extract Broth (CZB) | 5.8 | NaNO_3_ 3; KH_2_PO_4_ 1; MgSO_4_.7H_2_O 0.5; KCl 0.5; FeSO_4_.7H_2_O 0.01; Glucose 30 |
| Goose and Tschessch Broth (GTB) | 7.0 | Peptone 2; Glucose 10; MgSO_4_.7H_2_O 0.5; KH_2_PO_4_ 0.5 |
| Leonine Broth (LEB) | 7.0 | Peptone 0.625; Maltose 6.25; Malt extract 6.25; KH_2_PO_4_ 1.25, MgSO_4_.7H_2_O 0.625 |

**Fig. S1:** Fungal crude extract (TPEE) induced cell death in HeLa cells determined by PI staining (A) Untreated cells (B) treated with 10 µg/mL of fungal crude extract (B) treated with 50 µg/mL of fungal crude extract (C) treated with 100 µg/mL of fungal crude extract

**Fig. S2:** Fungal crude extract (TPEE) induced mitochondrial depolarization in HeLa cells determined by JC-1 staining (A) Untreated cells (B) positive control (treated with 200µM 2,4 DNP)(C) treated with 10 µg/mL of fungal crude extract (D) treated with 50 µg/mL of fungal crude extract (E) treated with 100 µg/mL of fungal crude extract

**Fig. S3:** Effect of fungal crude extract (TPEE) in ROS generation in HeLa cells determined by DCFH-DA staining (A) Untreated cells (B) positive control (treated with 800 µM H_2_O_2_) (C) treated with 10 µg/mL of fungal crude extract (D) treated with 50 µg/mL of fungal crude extract (E) treated with 100 µg/mL of fungal crude extract

**Fig. S4:** Concentration dependent *in vitro* DPPH scavenging activity of secondary metabolites produced by *T. purpureogenus.* The fungus was grown in various culture media for different time period and organic extracts of the total fungal culture were tested for their potential antioxidative activity by DPPH radical scavenging assay (A) Time dependent DPPH scavenging of fungal crude extract of *T. purpureogenus.* The fungus was grown in PDB for indicated time period and the ethyl acetate extract was tested for antioxidative activity (B) *In vitro* DPPH scavenging activity of different solvent extracts of *T. purpureogenus.* The fungus was grown in PDB for 21 days and different solvent extracts were tested for antioxidative activity (C) Effect of different culture media on the production of antioxidative secondary metabolites by *T. purpureogenus* . The fungus was grown in different media for 21 days and the hexane extracts (TPHE) were tested for antioxidative activity (D) Effect of NaCl on the production of antioxidant secondary metabolites by *T. purpureogenus.* The fungus was grown in MEB with different concentration of NaCl for 21 days and the hexane extracts (TPHE)were tested for antioxidative activity. Values are the means of three replicates±SD of three replicates.
